# Transcranial Direct Current Stimulation (tDCS) to Improve Gait in Multiple Sclerosis: A Timing Window Comparison

**DOI:** 10.3389/fnhum.2019.00420

**Published:** 2019-11-28

**Authors:** Craig D. Workman, John Kamholz, Thorsten Rudroff

**Affiliations:** ^1^Department of Health and Human Physiology, University of Iowa, Iowa City, IA, United States; ^2^Department of Neurology, Carver College of Medicine, University of Iowa Hospitals and Clinics, Iowa City, IA, United States

**Keywords:** transcranial direct current stimulation (tDCS), multiple sclerosis, gait, neuromodulation, 6-min walk test

## Abstract

Unilateral weakness of the lower limb is a hallmark of multiple sclerosis (MS) and a significant contributor to the progressive worsening of walking ability. There are currently no effective rehabilitation strategies targeting strength asymmetries and/or gait impairments in people with MS (PwMS). Transcranial direct current stimulation (tDCS) has improved motor outcomes in various populations, but the effect of tDCS on gait in PwMS and the ideal timing window of tDCS application are still unknown. This study investigated the effects of tDCS, either before or during a 6 min walk test (6MWT), on the distance walked and gait characteristics in PwMS. Twelve participants were recruited and randomly assigned into BEFORE or DURING groups (both *n* = 6). The BEFORE group received stimulation before performing a 6MWT (sham/2 mA, 13 min). The DURING group received stimulation only during a 6MWT (sham/2 mA, 6 min). Stimulation was over the more MS-affected primary motor cortex (M1). Distance walked and gait characteristics of the walk were the primary and secondary outcomes. The results indicated a significant decrease in distance walked in the DURING group (*p* = 0.026) and a significant increase in gait velocity in the BEFORE group (*p* = 0.04). These changes were accompanied by trends (*p* < 0.1) in distance walked, gait velocity, and stride length. Overall, the results of this study suggest that tDCS performed before a 6MWT might be more effective than tDCS during a 6MWT and that a single session of tDCS may not be sufficient to influence gait.

**Clinical Trial Registration:** www.ClinicalTrials.gov, identifier #NCT03757819.

## Introduction

Weakness on one side of the body is a hallmark of multiple sclerosis (MS) and has been determined to be a significant contributor to the progressive worsening of walking abilities (Kent-Braun et al., 1997; Thoumie and Mevellec, 2002; Mevellec et al., 2003; Ng et al., 2004; Kalron et al., 2011; Broekmans et al., 2013). Currently, there are no efficient rehabilitation strategies targeting strength asymmetries and/or gait impairments in people with MS (PwMS). Many of the current treatments, including pharmaceuticals, are only mildly effective and are often very expensive. Thus, a practical, inexpensive, and effective adjunct treatment is required. One possible modality that fulfills these requirements is transcranial direct current stimulation (tDCS; Jeffery et al., 2007). tDCS uses small currents applied to the scalp to increase the excitability of cortical neurons by increasing their spontaneous firing rate (Jeffery et al., 2007).

tDCS has been consistently shown to enhance motor performance in healthy participants (Williams et al., 2013; Lima De Albuquerque, 2015; Kaminski et al., 2016; van Asseldonk and Boonstra, 2016), older adults (Hummel et al., 2010; Zimerman et al., 2013; Hardwick and Celnik, 2014; Poston et al., 2015), stroke patients (Jayaram and Stinear, 2009; Sohn et al., 2013; Au-Yeung et al., 2014), and people with Parkinson’s disease (Fregni et al., 2006; Benninger et al., 2010; Grüner et al., 2010; Poston et al., 2013). One study of tDCS in PwMS found that a single session of anodal tDCS (1 mA for 20 min) over the primary motor cortex (M1) contralateral to the more-impaired hand resulted in increased corticospinal output and projection strength compared to sham stimulation (Cuypers et al., 2013). However, other findings (Meesen et al., 2014) indicated that one tDCS session was not able to improve motor performance of the more-impaired hand of PwMS more than sham. Only one study has investigated a single application of anodal tDCS to improve knee extensor fatigability in PwMS and found no effect on an isometric task (Proessl et al., 2018). Although some have investigated the effects of multiple tDCS sessions on dynamic functional tasks, like gait, in PwMS (Oveisgharan et al., 2019) and in other neurological populations (de Paz et al., 2019), the results have been mixed. No study to date has investigated the effects of a single application of tDCS to improve gait in PwMS.

Importantly, there is also a lack of evidence regarding the best timing window for tDCS, as no studies have directly compared tDCS before with tDCS during a functional motor performance. tDCS applied to the resting motor cortex (before) may activate neuronal populations in a non-specific way that is unlike activation for a specific motor task (Nitsche et al., 2008). Thus, theoretically, tDCS during a motor task could lead to an improved motor performance because the stimulation may further enhance the normal increases in cortical excitability and synaptic efficiency in specific, task-related neural circuits active during task execution. On this topic, one study measured cortical excitability before, during, and after tDCS (Santarnecchi et al., 2014) and found that excitability only reliably increased after stimulation, but not during. In addition, most studies have applied tDCS before measuring various outcomes (Cuypers et al., 2013; Ferrucci et al., 2014; Saiote et al., 2014; Tecchio et al., 2015; Hanken et al., 2016; Chalah et al., 2017). However, tDCS has previously enhanced motor learning when applied during the learning process (Ammann et al., 2016). Thus, the ideal timing window for tDCS application remains ambiguous, especially considering the lack of comparative studies.

Therefore, the purpose of this study was to determine whether one application of tDCS over the M1 representation of the more MS-affected leg would increase the distance walked in a 6 min walk test (6MWT) in PwMS. An additional purpose was to determine if tDCS applied during a 6MWT would increase the distance walked compared to tDCS before a 6MWT. We hypothesized that, compared to sham, tDCS would increase distance walked and that tDCS during the 6MWT would increase distance walked to a greater extent than tDCS before the 6MWT in PwMS.

## Materials and Methods

### Participants

Twelve participants with relapsing-remitting MS were recruited (see Table 1 for demographic information). Inclusion criteria were: (1) medically diagnosed with MS; (2) 18–70 years of age; (3) moderate disability (i.e., a score of 2–6 on the Patient-Determined Disease State (PDDS) questionnaire); (4) self-reported differences in function between the legs; and (5) able to walk for 6 min. Exclusion criteria included: (1) relapse within the last 60 days; (2) changes in disease-modifying medications within the last 45 days; (3) concurrent neurological or neuromuscular disease; (4) hospitalization within the last 90 days; (5) diagnosed depression; and (6) inability to understand or sign the consent form. Participants were randomly assigned into two groups in a counterbalanced fashion. All participants signed informed consent and the study was approved by the Institutional Review Board at the University of Iowa. Study procedures were conducted in accordance with the Declaration of Helsinki.

**Table 1:** Subject demographic information.

	Before	During	All
Sex (M/F)	2/4	4/2	6/6
Age (years)	47.0 ± 13.6	55.8 ± 7.4	51.4 ± 11.4
Height (cm)	160.4 ± 5.4	177.4 ± 10.8	168.9 ± 12.0
Weight (kg)	66.7 ± 13.4	86.1 ± 22.2	76.4 ± 20.2
Time since diagnosis (years)	17.7 ± 12.9	17.5 ± 12.7	17.6 ± 12.2
PDDS	2.7 ± 1.4	3.8 ± 1.2	3.3 ± 1.4
FSS	4.2 ± 2.3	5.3 ± 0.5	4.8 ± 1.7
Medications		
DMT (%)	66.7	66.7	66.7
Others (%)	16.7	33.3	25.0

*Data are mean ± SD. Note: PDDS, Patient-Determined Disease Steps; FSS, Fatigue Severity Scale; DMT, disease modifying therapies (Aubagio, Ocrevus, Tecfidera, or Copaxone); Others, fatigue (Modafinil, Adderall), spasticity (Baclofen), or walking (Ampryra). FSS served as the covariate in the statistical analysis. Medications are percentage of subjects taking either DMT or other MS-related drugs*.

### Experimental Design

This study employed a double-blind, sham-controlled, randomized crossover design. Each participant attended three sessions. In the first session, participants were consented and completed the PDDS and Fatigue Severity Scale (FSS) questionnaires. Then, after a counter-balanced randomization into BEFORE or DURING groups (both *n* = 6), the participants completed a 6MWT for baseline/familiarization purposes. The second and third sessions involved tDCS or sham either before or during a 6MWT, depending on group assignment. The tDCS stimulation was randomly assigned to either session two or three, and sham was performed in the other session.

### Leg Strength, 6MWT, and tDCS

Isokinetic maximal voluntary contractions (MVCs) of the right and left knee extensors were performed to determine the more-affected leg. When strength differences were less than 10% (Sapega, 1990), the more-affected leg was based on the participants’ self-report. For the 6MWT (Goldman et al., 2008; Socie et al., 2014; McLoughlin et al., 2016), participants walked back and forth between two markers spaced 30 m apart for 6 min. The distance walked was the primary outcome measure. Additionally, because we expected that tDCS would alter the utilization of the more-affected leg, standard gait characteristics (e.g., gait speed, cadence, and stride length) for the 6MWT were also collected as secondary outcomes using a six sensor OPAL system (APDM Inc., Portland, OR, USA) for both tDCS and sham sessions (Washabaugh et al., 2017).

A tDCS device (ActivaDose II, ActivaTek Inc., Salt Lake City, UT, USA) delivered a small direct current through two sponge surface electrodes (5 cm × 5 cm, soaked with 15 mM NaCl). The anode was located over the motor cortex representation of the more-affected leg (C3 or C4 on the International EEG System) and the cathode was placed on the contralateral supraorbital area (Au-Yeung et al., 2014). The electrodes were held in place with a Caputron Universal tDCS Strap (Caputron, New York City, NY, USA). When stimulation was applied in the 6MWT for the DURING group, the device was secured to the middle of the upper back of the participant using the OPAL sensor chest strap. None of participants reported any discomfort from having the device secured in this fashion. Active tDCS involved a 30 s ramp-up to 2 mA, after which the intensity stayed at 2 mA for the duration of the stimulation period. At the end of the stimulation time, the intensity was ramped-down to 0 mA over 30 s. For sham, participants experienced the initial 30 s ramp-up to 2 mA, after which the intensity was ramped-down to 0 mA for the remainder of the stimulation period.

For the BEFORE group, stimulation was applied for 13 min while seated comfortably in a chair. Thirteen minutes of tDCS results in after-effects sufficient to increase excitability for the duration of the 6MWT (Nitsche and Paulus, 2001). Immediately after the stimulation, the participants completed the 6MWT as described above. For the DURING group, stimulation was applied only during the 6MWT (i.e., for 6 min), which has been shown to be sufficient to induce changes in cortical excitability (Nitsche and Paulus, 2000, 2001; Santarnecchi et al., 2014; Buch et al., 2017). Because a purpose of this study was to investigate two different tDCS timing windows, it was necessary to include tDCS only during the 6MWT and not a combination of before and during. However, previous studies have indicated that short duration stimulation (i.e., 5 min) results in a rapid decline in excitability toward baseline levels (≤5 min; Nitsche and Paulus, 2000). Therefore, a relatively longer stimulation time for the BEFORE group (i.e., 13 min) was necessary to induce excitability increases to the same level as the 6 min duration, but also sufficient to last through the completion of the 6MWT (Nitsche and Paulus, 2001). Nevertheless, the intensity of tDCS was 2 mA for both groups (Nitsche and Paulus, 2000; Meesen et al., 2014). The participants were blind to which stimulation condition they experienced and test administrators that measured the distance walked were also blind to stimulation condition (i.e., double-blind). To determine blinding integrity, participants were asked to guess which stimulation condition (tDCS or sham) they experienced at the end of sessions two and three. Feedback about the accuracy of guesses was not provided until the participant had completed all experimental conditions.

### Statistical Analysis

The distance walked during 6MWT and gait characteristics were analyzed with a repeated-measures ANCOVA, with stimulation (tDCS vs. sham) as a within-subjects factor and group (BEFORE vs. DURING) as a between-subjects factor. Additionally, to control for the individual baseline fatigue of the participants, FSS score was input as a covariate. Paired *t*-tests clarified significant main and interaction effects. Significance was accepted at *p* < 0.05, after Bonferroni correction. All analyses were performed using SPSS 25 (IBM Corp., Armonk, NY, USA). The percentage of correct guesses in each stimulation condition (tDCS or sham) assessed blinding integrity. Data are reported as mean ± SD in tables and as mean ± SE in figures.

## Results

**Figure 1:**
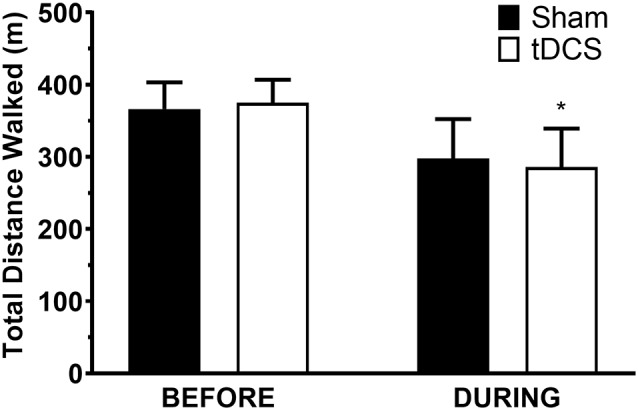
Distance walked in the 6-min walk test. Data are mean ± SEM. *Indicates significantly different from sham.

**Figure 2:**
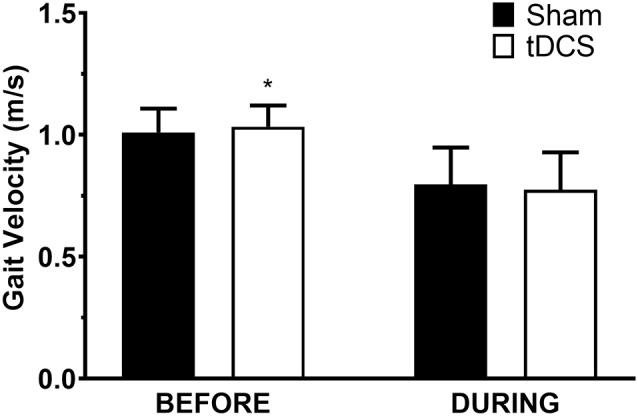
Gait velocity in the 6-min walk test. Data are mean ± SEM. *Indicates significantly different from sham.

All participants completed all scheduled sessions. The results of the analysis indicated no significant main effects of stimulation or group (*p* > 0.05), but there were significant stimulation*group interactions for total distance walked (*p* = 0.011), gait velocity (*p* = 0.014), more-affected stride length (*p* = 0.023), and less-affected stride length (*p* = 0.032). Pairwise tests further indicated that DURING walked a significantly shorter distance in tDCS compared with sham (mean difference (95% confidence interval); −14.6 m (−27.0 to −2.2), *p* = 0.026; Figure 1) accompanied by decreases in velocity and stride length on the less-affected side that approached significance [−0.25 m/s (−0.051 to −0.001), *p* = 0.06 and −0.21 m/s (−0.046 to −0.003), *p* = 0.08, respectively]. BEFORE had a significant increase in gait velocity in tDCS compared with sham [0.03 m/s (>0.000–0.053), *p* = 0.04; Figure 2] accompanied by nearly significant increases in distance walked [11.1 m (−1.319 to 23.445); *p* = 0.07] and stride length on the more-affected side [0.02 m (−0.001 to −0.042, *p* = 0.06]. See Table 2 for other paired test results. In addition, the same percentage of subjects correctly guessed the tDCS and sham conditions (both 83.3%).

**Table 2:** Analysis results for the gait characteristics during the 6-min walk test.

	Before			During		
	Sham	tDCS	*p*	Sham	tDCS	*p*
Distance (m)	366.3 ± 90.2	375.0 ± 77.8	0.07	298.0 ± 133.1	285.7 ± 131.0*	0.02
Velocity (m/s)	1.01 ± 0.23	1.03 ± 0.21*	0.04	0.80 ± 0.37	0.78 ± 0.37	0.06
Cadence (str/min)	110.5 ± 9.4	111.6 ± 8.0	0.25	92.6 ± 16.4	91.8 ± 5.7	0.38
Stride Length (m)						
More-affected	1.09 ± 0.19	1.11 ± 0.17	0.06	0.98 ± 0.34	0.97 ± 0.35	0.09
Less-affected	1.08 ± 0.18	1.09 ± 0.16	0.11	0.98 ± 0.35	0.96 ± 0.36	0.08

Data are mean ± SD. Note: results are after controlling for Fatigue Severity Scale score and Bonferroni correction. Str, strides. *Indicates significant difference between sham and tDCS.

## Discussion

The purpose of this study was to determine whether one application of tDCS over the M1 representation of the more MS-affected leg would increase the distance walked in a 6MWT in PwMS and to determine if tDCS applied during a 6MWT would increase the distance walked compared to tDCS before a 6MWT. The results indicated that tDCS increased gait velocity in the BEFORE group only, which coincided with trending changes in distance walked and stride length on the more-affected side (Table 2). However, contrary to our second hypothesis that distance walked would be greater in DURING, this group walked a significantly shorter distance with tDCS, accompanied by trending decreases in velocity and stride length on the less-affected side that approached significance (Table 2). Taken together, and considering the trends toward significance (i.e., *p* ≤ 0.1) that similarly reflected the distance walked and velocity changes of both groups, these results indicate that tDCS before a gait task might be more effective than tDCS during a gait task.

Interestingly, the effect of tDCS on the DURING group was an overall decrease in 6MWT performance. This is in contrast to previous studies that found significant enhancing effects of tDCS during motor learning (Ammann et al., 2016). However, an important distinction between the studies reviewed by Ammann et al. (2016) and the current study is the purpose of the stimulation. Studies on motor learning used tDCS during a task (often called “online”) to enhance the acquisition of a novel skill; here, tDCS was used to affect the motor performance of a presumably well-learned, everyday task. Thus, the difference between the acquisition of a new skill and the performance of a learned task may represent an important discrepancy between the effects of tDCS during a task. Furthermore, a previous study (Santarnecchi et al., 2014) investigating the time course of the effects of tDCS on cortical excitability found that excitability changes were ambiguous during the stimulation (with the exception of an excitability increase at min 2.5), but had consistent significant increases for several minutes after stimulation (Santarnecchi et al., 2014), which agrees with the data of the current study. Some have also suggested that the cortex may try to maintain an excitability homeostasis, even in a diseased state (Chai et al., 2019). Thus, in populations like MS that have reduced baseline excitability (Zipser et al., 2018), performing tDCS during gait might interfere with endogenous gait signals and the homeostatic maintenance efforts of the cortex. More investigation into the difference between the effects of tDCS during skill acquisition and during a motor performance, as well as the mechanisms underpinning these differences, is certainly warranted.

In addition, many motor learning paradigms involve relatively simple movements of the digits or upper extremity (Ammann et al., 2016), while gait requires complex joint movements and coordination of multiple motor and sensory systems (Bollens et al., 2014). Likewise, a recent review (Machado et al., 2019) analyzed the results of tDCS studies on muscular strength and muscular endurance. The authors found that 66.7% of studies on muscular strength and 50% of studies on muscular endurance reported significant improvements from tDCS. However, most of these studies investigated joints in isolation (i.e., using a dynamometer), which makes the complexity of those tasks necessarily less than walking. Therefore, task complexity, and or specificity, may also explain the conflicting findings encountered in many tDCS investigations on motor performance.

The different stimulation times between BEFORE and DURING may also represent another important factor. Anodal tDCS for 5 min has previously resulted in increased cortical excitability (Nitsche and Paulus, 2000, 2001; Santarnecchi et al., 2014; Buch et al., 2017), but these increases had shorter latencies than longer stimulation times (i.e., 13 min; Nitsche and Paulus, 2001). Therefore, even though cortical excitability may have increased with 6 min of stimulation, it is possible that the short stimulation period for the DURING group may have been insufficient to influence the performance of the 6MWT. On the contrary, the stimulation time of the BEFORE group was closer to previous studies that have reported performance improvements with tDCS in other populations (Benninger et al., 2010; Hummel et al., 2010; Au-Yeung et al., 2014; van Asseldonk and Boonstra, 2016) and was accompanied by increased gait velocity. Therefore, the results of the present study may suggest that a certain (minimum) stimulation time may need to be reached before performance improvements become evident.

Similarly, the effect of a single session of tDCS may also be inadequate to induce motor performance changes (Meesen et al., 2014; Proessl et al., 2018). In particular, considering that MS has presumably been affecting and driving motor adaptations/maladaptations for years, it may be unrealistic to expect large effects from one acute application of tDCS (Sadnicka et al., 2014). Indeed, the significant increase in velocity found in the BEFORE group may not be clinically relevant, but the accompanying trending increase in distance walked (~11 m) may indicate clinically significant changes. To this point, there is evidence that multiple sessions of tDCS may result in greater performance outcomes (Workman et al., 2019), even during a motor task (Dumel et al., 2016; Ho et al., 2016; Yosephi et al., 2018). Thus, investigation of repeated applications of tDCS, both before and during, to influence gait in PwMS is justified.

The most prominent limitation of this study is the relatively small number of participants in each group, which suggests caution in generalizing the results. In addition, the differences in sex, height, weight, or disability (PDDS) could have contributed to the lack of significant group effects. There was also no assessment of cortical excitability with transcranial magnetic stimulation (TMS), which would have provided information on the excitability changes from tDCS in the BEFORE and DURING groups. Additionally, changes in the ratings of perceived exertion (RPE) in the different conditions were not measured. A different RPE in tDCS compared with sham, or between the two groups after the 6MWT, would have helped determine if PwMS experienced changes in perceived exertion and further clarified the clinical relevance of the results. Additionally, the stimulation montage (i.e., unilateral M1) used may represent another limitation. Previous studies using similar montages have had yielded positive results (Cogiamanian et al., 2007; Kan et al., 2013). However, there is evidence that bilateral montages (i.e., targeting both hemispheres) may be superior to unilateral montages (Naros et al., 2016; Angius et al., 2018; Cancelli et al., 2018). Additionally, cerebellar montages may be preferable in gait investigations because the cerebellum controls several important aspects of gait (Thach and Bastian, 2004). In addition, the relatively large size of the stimulation electrodes may have influenced brain areas surrounding M1 (Bastani and Jaberzadeh, 2013; Ho et al., 2016), which could also have influenced performance.

Future studies should continue to assess the effects of tDCS on complex motor performances and explore the ideal window for applying tDCS, especially in multiple sessions. Future work should also investigate the mechanisms underpinning motor performances and would benefit from TMS (cortical excitability), electromyography (muscle activation), and positron emission tomography (PET) or other brain activity measures. Furthermore, explorations and comparisons of different task complexities, different montages (i.e., bilateral, cerebellar), and traditional vs. high definition tDCS (HD-tDCS) focal stimulation (Reckow et al., 2018) will further refine tDCS applications aimed at ameliorating the symptoms of debilitating neurological conditions.

## Summary

The BEFORE group performed the 6MWT at a higher gait velocity after tDCS and the DURING group walked a significantly shorter distance in the 6MWT with tDCS. These changes were accompanied by trends (*p* < 0.1) in distance walked, gait velocity, and stride length in the same direction as the significant results for each group. Overall, the results of this study suggest that tDCS performed before a 6MWT might be more effective than tDCS during a 6MWT and that a single session of tDCS may not be sufficient to influence gait.

## Data Availability Statement

The datasets generated for this study are available on request to the corresponding author.

## Ethics Statement

The studies involving human participants were reviewed and approved by the Institutional Review Board at the University of Iowa. Study procedures were conducted in accordance with the Declaration of Helsinki. The patients/participants provided their written informed consent to participate in this study.

## Author Contributions

CW and TR designed the study. Participants were recruited by JK and CW. CW performed data collection and analysis, and CW and TR interpreted the results. The manuscript was drafted by CW and TR and JK critically revised the manuscript. All authors approved the final version.

## Conflict of Interest

The authors declare that the research was conducted in the absence of any commercial or financial relationships that could be construed as a potential conflict of interest.
